# Lebensqualität nach innerklinischem Herz-Kreislauf-Stillstand: 11-Jahres-Ergebnisse einer Universitätsklinik

**DOI:** 10.1007/s00101-024-01423-3

**Published:** 2024-05-31

**Authors:** Benedikt Treml, Christine Eckhardt, Christoph Oberleitner, Thomas Ploner, Christopher Rugg, Aleksandra Radovanovic Spurnic, Sasa Rajsic

**Affiliations:** 1 Universitätsklinik für Anästhesie und Intensivmedizin, Medizinische Universität Innsbruck, Anichstraße 35, 6020 Innsbruck, Österreich; 2 Universitätsklinik für Innere Medizin, Medizinische Universität Innsbruck, 6020 Innsbruck, Österreich; 3 Klinik für Infektions- und Tropenkrankheiten, Universität Klinisches Zentrum Serbiens, 11000 Belgrad, Serbien

**Keywords:** Innerklinischer Herz-Kreislauf-Stillstand, Überleben, EQ-5D-5L, Kardiopulmonale Reanimation, Risikofaktoren, Mortalität, In-hospital cardiac arrest, Survival, EQ-5D-5L, Cardiopulmonary resuscitation, Risk factors, Mortality

## Abstract

**Hintergrund:**

Ein Herz-Kreislauf-Stillstand (HKS) ist ein lebensbedrohlicher Zustand, der weltweit eine der häufigsten Todesursachen darstellt. Die Literatur bezüglich der Lebensqualität nach kardiopulmonaler Reanimation ist limitiert und beinhaltet hauptsächlich Daten von HKS außerhalb des Krankenhauses. Diese könnten sich bezüglich Epidemiologie und Outcome von innerklinischen Herz-Kreislauf-Stillständen (IHCA) unterscheiden. Ziel dieser Studie war es, die Lebensqualität mittels EQ-5D-5L-Fragebogen nach einem IHCA zu untersuchen und mögliche Risikofaktoren für ein schlechteres Outcome zu ermitteln.

**Material und Methoden:**

Diese retrospektive Datenanalyse und prospektive Erhebung der Lebensqualität umfasste alle Patient:innen, die einen IHCA im Zeitraum von 2010 bis 2020 überlebten. Der primäre Endpunkt der Studie war die Lebensqualität am Stichtag nach einem IHCA. Sekundäre Endpunkte umfassten Prädiktoren für ein schlechteres Outcome.

**Ergebnisse:**

Insgesamt wurden innerhalb des Zeitraums von 11 Jahren 604 innerklinische Reanimationen durchgeführt, wobei 61 (10 %) der Patient:innen bis zum Zeitpunkt der Befragung überlebten. Achtundvierzig (79 %) Patient:innen erfüllten die Einschlusskriterien, und 31 (65 %) wurden in diese Studie eingeschlossen. Es gab keinen signifikanten Unterschied in der Lebensqualität vor und nach dem HKS (EQ-5D-5L Utilität 0,79 vs. 0,78; *p* = 0,567) und im EQ-5D-5L-VAS-Score. Eine chirurgische Indikation für die Krankenhausaufnahme war mit einer besseren Lebensqualität nach dem IHCA assoziiert, verglichen mit einer medizinischen Aufnahmeindikation (*p* = 0,009).

**Schlussfolgerung:**

Patient:innen, die einen innerklinischen Herz-Kreislauf-Stillstand überlebten, zeigten eine vergleichbare Lebensqualität vor und nach dem Ereignis. Dennoch berichteten die Patient:innen über eine Verschlechterung der Mobilität und der Angst/Depression. Künftige Studien sollten bei der Erhebung der Folgen eines Herz-Kreislauf-Stillstands die verfügbaren Instrumente zur Bewertung der Lebensqualität miteinbeziehen.

**Zusatzmaterial online:**

Die Online-Version dieses Beitrags (10.1007/s00101-024-01423-3) enthält weitere Abbildungen und Tabellen.

## Abstract (en)

**Background:**

Cardiac arrest is a life-threatening condition requiring urgent medical care and is one of the leading causes of death worldwide. Given that in-hospital cardiac arrest (IHCA) is still poorly investigated, data on health-associated quality of life thereafter remains scarce. The available evidence is mostly transferred from out-of-hospital cardiac arrest studies, but the epidemiology and determinants of success might be different. The aim of the study was to investigate the change in the quality of life after in-hospital cardiac arrest and to identify potential risk factors for a poor outcome.

**Material and methods:**

This retrospective analysis of data and prospective evaluation of quality of life included all patients surviving an IHCA and being treated by the emergency medical team between 2010 and 2020. The primary endpoint of the study was the quality of life after IHCA at the reference date. Secondary endpoints covered determination of risk factors and predictors of poor outcome after in-hospital cardiopulmonary resuscitation.

**Results:**

In total 604 patients were resuscitated within the period of 11 years and 61 (10%) patients survived until the interview took place. Finally, 48 (79%) patients fulfilled the inclusion criteria and 31 (65%) were included in the study. There was no significant difference in the quality of life before and after cardiac arrest (EQ-5D-5L utility 0.79 vs. 0.78, *p* = 0.567) and in the EQ-5D-5L visual analogue scale (VAS) score.

**Conclusion:**

The quality of life before and after IHCA in survivors was good and comparable. The quality of life was mostly affected by reduced mobility and anxiety/depression. Future studies with larger patient samples should focus on potentially modifiable factors that could prevent, warn, and limit the consequences of in-hospital cardiac arrest. Moreover, research on outcomes of IHCA should include available tools for the quality of life assessment.

## Hintergrund und Fragestellung

Ein Herz-Kreislauf-Stillstand (HKS) ist zweifellos ein lebensbedrohlicher Zustand, der eine dringende medizinische Versorgung erfordert und weltweit eine der häufigsten Todesursachen darstellt [1]. Die Inzidenz eines innerklinischen Herz-Kreislauf-Stillstandes (IHCA) liegt in Europa bei 1,5 bis 2,8 Ereignissen/1000 Krankenhauseinweisungen. Die Überlebensrate, als wichtigster Outcome-Parameter, beträgt nach IHCA 12 % bis maximal 25 % [2–4]. Darüber hinaus soll das Überleben mit möglichst gutem neurologischen Outcome vergesellschaftet sein. Dazu können der neurologische Zustand mithilfe der „Cognitive Performance Category (CPC) Scale“ bzw. die Selbstständigkeit der Patient:innen und die Aktivitäten des täglichen Lebens z. B. mit der modifizierten Rankin Skala abgeschätzt werden [5]. Seit dem Statement des Liaison Committee on Resuscitation (ILCOR) aus dem Jahr 2018, welches die Vereinheitlichung der Outcome Parameter nach einem HKS zum Ziel hatte, rückte die Lebensqualität als zusätzlicher Parameter zur Prognoseabschätzung in den Fokus. Diese Empfehlung der ILCOR beinhaltet in ihrem „Core Outcome Set“, zusätzlich zur Erhebung der Überlebensrate und des neurologischen Zustands, die Bestimmung der gesundheitsbezogenen Lebensqualität [6]. Jahre später existiert dazu jedoch nach wie vor eine überschaubare Evidenz [7–19]. Der Großteil der Daten zur Lebensqualität nach kardiopulmonaler Reanimation stammt aus Studien zum OHCA, wobei unterschiedliche ätiologische und epidemiologische Faktoren, nicht nur für die Überlebensrate, sondern auch für die Lebensqualität entscheidend sein könnten [11–24]. Vergleiche von Lebensqualität von und nach IHCA sind spärlich [9–11].

Die Ziele dieser Studie waren einerseits die Erhebung der gesundheitsbezogenen Lebensqualität mithilfe des EQ-5D-5L bei IHCA-Patient:innen vor und nach dem Ereignis sowie die Evaluierung von möglichen Risikofaktoren für ein schlechteres Outcome.

## Studiendesign und Untersuchungsmethoden

In diese retrospektive Datenanalyse mit Erhebung der Lebensqualität vor und nach einem IHCA wurden Patient:innen eingeschlossen, welche zwischen Januar 2010 und Dezember 2020 einen IHCA erlitten und folgende Einschlusskriterien erfüllten: Überleben bis zum Stichtag der Lebensqualitätevaluierung (14.09.2021), Alter > 18 Jahre und aktueller Wohnsitz zum Stichtag in Österreich.

Diese Arbeit wurde entsprechend der Erklärung Strengthening the Reporting of Observational Studies in Epidemiology (STROBE, Zusatzmaterial online: Tabelle E1) und in Übereinstimmung mit der Konsenserklärung des International Liaison Committee on Resuscitation (ILCOR) verfasst [25]. Diese Studie wurde von der Ethikkommission der Medizinischen Universität Innsbruck, Österreich genehmigt (1151/2021).

### Zielsetzungen

Das primäre Ziel dieser Studie war die Evaluierung der gesundheitsbezogenen Lebensqualität mittels EQ-5D-5L-Fragebogen vor und nach einem IHCA [27]. Zudem sollten Prädiktoren und Risikofaktoren für ein schlechteres Outcome nach einem IHCA ermittelt werden.

### Auswahl der Patient:innen

Alle Patient:innen, die im Zeitraum zwischen Januar 2010 und Dezember 2020 einen IHCA im Universitätsklinikum der Medizinischen Universität Innsbruck erlitten und überlebten, wurden schriftlich über eine mögliche Studienteilnahme informiert. Nach Einholen der Einverständniserklärung wurde den Studienpatient:innen 2 Exemplare des EQ-5D-5L-Fragebogen zu Evaluierung der gesundheitsbezogenen Lebensqualität vor und nach einem IHCA zugeschickt.

### Datenerhebung

Die retrospektive Datenextraktion umfasste folgende Parameter:

1. soziodemografische Eigenschaften, den Simplified Acute Physiology Score III (SAPS III) und den Charlson-Komorbiditäts-Score, die Erkrankung, die zur Hospitalisierung führte, die kardiopulmonale Reanimation in der Vorgeschichte, detaillierte Informationen über Komorbiditäten, die Einnahme verschiedener Medikamente und Vitalparameter von 48 h und 24 h vor dem HKS;
2. Umstände des HKS, Einzelheiten der Intervention des Notfallteams, Werte der ersten Blutgasanalyse und den nachfolgenden Analysen während des Aufenthalts auf der Intensivstation.

Prospektiv wurde die Lebensqualität mittels EQ-5D-5L erhoben. Hierfür erhielten die Studienteilnehmer:innen 2 Versionen des Fragebogens und wurden gebeten, den Fragebogen einmal rückblickend, dem Zustand vor dem IHCA, und einmal dem aktuellen Zustand entsprechend dem Zustand nach dem IHCA, auszufüllen. Zur Erhöhung der Rücklaufquote wurden die Patient:innen mehrmalig kontaktiert, das Porto für die Rückensendung wurde übernommen sowie telefonische Hilfestellung beim Ausfüllen des Fragebogens angeboten.

### Erhebung der Mortalität

Die Mortalität wurde zum Stichtag des Versendens der Lebensqualitätfragebogen erhoben.

### Behandlung der Patient:innen mit einem IHCA

Die Notfallteams, die rund um die Uhr für die Versorgung der IHCA verantwortlich sind, (strukturierte Alarmkriterien „Single Track and Trigger System“ laut ERC-Leitlinien) bestanden aus mindestens einem Intensivmediziner und 2 Intensivpflegekräften [26]. Je nach Ort des Notfalls wurde ein Notfallteam entweder von der anästhesiologischen oder der internistischen Intensivstation entsandt. Nach der Reanimation wurden alle Patient:innen auf einer der Kategoie-3-Intensivstationen der Univ.-Klinik für Anästhesiologie und Intensivmedizin oder der Inneren Medizin der Medizinischen Universität Innsbruck, Österreich, behandelt.

### Gesundheitsbezogene Lebensqualität

Der EQ-5D-5L ist ein validierter, patientenorientierter Fragebogen, welcher die gesundheitsbezogene Lebensqualität in 5 Dimensionen evaluiert [27]. Diese beinhalten Mobilität, Selbstversorgung, Schmerzen/Beschwerden, gewohnte Aktivität und Angst/Depression in einem Punktebewertungssystem, wovon sich der EQ-Index-Wert ableitet [27, 28]. In jeder Dimension sind 5 Stufen zur Beschreibung der Lebensqualität vorgesehen: keine Probleme, leichte Probleme, mäßige Probleme, schwere Probleme und extrem/unmöglich. Zudem erhebt dieser Fragebogen eine visuelle Analogskala (VAS), die von 0 bis 100 reicht, wobei 0 den Tod und 100 den bestmöglichen Gesundheitszustand bedeuten.

### Statistik

Die statistischen Analysen wurden mit IBM SPSS (Version 22.0, freigegeben 2013; Fa. IBM Corp., Armonk, NY, USA) durchgeführt. Es wurde ein Signifikanzniveau von *p* < 0,05 angewendet, und alle statistischen Auswertungen waren zweiseitig. Die Ergebnisse werden als Mittelwert (Standardabweichung), Häufigkeit (in Prozent) und Median (Bereich, Minimum-Maximum) dargestellt. Ein *t*-Test für unabhängige Stichproben und Mann-Whitney-U-Test wurden entsprechend der Normalverteilung angewendet. Assoziationsanalysen wurden mittels Chi-Quadrat-Test und Exakter Fishers-Test erhoben. Anhand der Differenz des EQ-Index vor und nach dem IHCA wurden die Patient:innen in 2 Gruppen eingeteilt: Patient:innen mit einer Verbesserung (alle Patient:innen mit gleicher oder positives Delta im EQ-Index) und Patient:innen mit einer Verschlechterung der Lebensqualität (alle Patient:innen mit negativer Differenz im EQ-Index).

## Ergebnisse

**Abb. 1:**
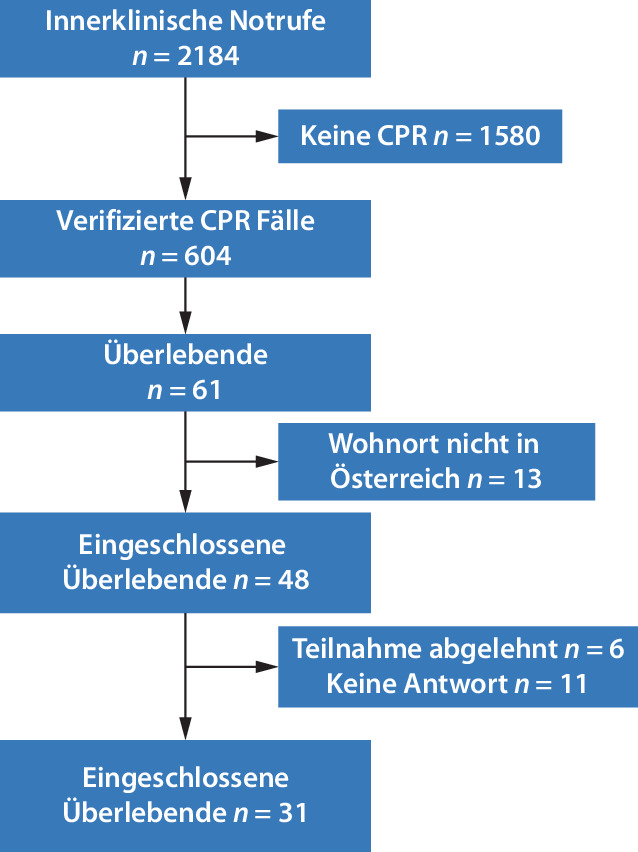
Flussdiagramm: Studiendesign. *CPR* kardiopulmonale Reanimation

In einem Zeitraum von 11 Jahren wurden 2184 innerklinische Notfalleinsätze durchgeführt, von denen 645 auf einen HKS zurückzuführen waren. Bei 604 Patient:innen wurde eine kardiopulmonale Reanimation eingeleitet, bei 44 Patient:innen wurde aufgrund einer aufrechten „Do-not-resuscitate“-Anweisung auf eine Reanimation verzichtet. Von allen reanimierten Patient:innen waren 61 (10 %) am 14.09.2021 am Leben. Zwischen dem IHCA und der ersten Kontaktaufnahme im Rahmen der Studie vergingen zwischen 9 und 140 Monate. Von insgesamt 61 Patient:innen erfüllten 48 (79 %) die Einschlusskriterien. Von 48 versandten Fragebogen wurden 31 (65 %) komplett ausgefüllt und konnten in die Studienauswertung eingeschlossen werden. Elf Patient:innen gaben trotz Nachfrage keine Rückantwort. Die Rücklaufquote betrug somit 64,5 % (Abb. 1).

Die demografischen und klinischen Patientencharakteristika sind in Tab. 1 und 2 zusammengefasst.

**Tab. 1:** Demografie und medizinische Vorgeschichten der eingeschlossenen Patient:innen (*n* = 31)

Patient:innen-Charakteristika	*n* (%)
Alter (Jahre)	66,2 ± 12,3
Männliches Geschlecht	21 (67,7)
Body-Mass-Index (kg/m^2^)	27,4 ± 6,9
Präklinischer Herz-Kreislauf-Stillstand	4 (12,9)
Krankenhausaufenthalt (Tage)	16 (2–335)
ICU-Aufenthalt (Tage)	2 (0–20)
*Klinische Scores*	
SAPS III Score	51,2 ± 13,1
Charlson Comorbidity Index	4,2 ± 2,4
*Krankenhausaufnahme*	
Ambulant/nicht stationär	3 (9,7)
Stationär	24 (77,4)
Notfallaufnahme	4 (12,9)
*Dauermedikation*	
β‑Blocker	14 (45,2)
ACE-Hemmer	9 (29,0)
Antiarrhythmika	1 (3,2)
Antikoagulation	12 (38,7)
Antidiabetika	3 (9,7)
Chemotherapie	1 (3,2)

Die Daten sind angegeben als Mittelwert ± Standardabweichung, Median (Minimum bis Maximum) oder Anzahl (*n*) der Patient:innen (Anteil [%] in Klammern)

*ACE* „angiotensin-converting enzyme“, *SAPS* Simplified Acute Physiology Score, *ICU* Intensivstation

**Tab. 2:** Medizinische Vorgeschichten der eingeschlossenen Patient:innen (*n* = 31)

Komorbiditäten und Risikofaktoren	*n* (%)
Rauchen	9 (29,0)
Arterielle Hypertonie	24 (77,4)
Dyslipidämie	12 (38,7)
Koronare Herzkrankheit	16 (51,6)
Vorheriger Myokardinfarkt	7 (22,6)
Vorherige Arrhythmien	3 (9,7)
Vorhofflimmern	8 (29,0)
Kongestive Herzinsuffizienz	0 (0,0)
Herzoperation in Vorerkrankungen	7 (22,6)
Reanimation in Vorerkrankungen	4 (12,9)
Pulmonalembolie	3 (9,7)
pAVK	3 (9,7)
Zerebrovaskuläre Erkrankung	6 (19,4)
Chron. Lungenerkrankung	2 (6,5)
Diabetes mellitus (DM)	4 (12,9)
DM mit Endorganschaden	0 (0,0)
Chronische Lebererkrankung	1 (3,2)
Chronische Nierenerkrankung	4 (12,9)
Chronische Dialyse	0 (0,0)
Malignität/Tumor	6 (19,4)

Die Daten sind angegeben als Anzahl (*n*) der Patient:innen (Anteil [%] in Klammern). Zerebrovaskuläre Erkrankung umfassen Schlaganfall, transiente ischämische Attacke und intrakranielle Blutung, Lungenerkrankungen umfassen Asthma und chronisch obstruktive Lungenerkrankung

*pAVK* periphere arterielle Verschlusskrankheit

Von 28 (90 %) beobachteten IHCA waren 16 (52 %) Patient:innen vor und während des IHCA monitorisiert. Hierbei wurde bei 9 (60 %) Patient:innen vor dem IHCA ein Sinusrhythmus, bei 3 (27 %) ein funktionierender Herzschrittmacherrhythmus und bei 2 Patient:innen Vorhofflimmern aufgezeichnet. Bei 29 (94 %) Patient:innen wurde beim Eintreffen des Notfallteams eine Herzdruckmassage und bei 18 (58 %) Patient:innen wurde eine automatische externe Defibrillation durchgeführt. Kammerflimmern oder Kammertachykardie waren die häufigsten dokumentierten initialen EKG-Rhythmen, gefolgt von 6 (21 %) Patient:innen mit einer Asystolie. Brustschmerzen wurden bei 10 (32 %) Patient:innen kurz vor dem Notruf angegeben. Es gab keine Unterschiede in den dokumentierten Vitalparametern (Körpertemperatur, Herzfrequenz und Sauerstoffsättigung im Blut) oder in den Bedingungen vor dem Herz-Kreislauf-Stillstand, Tab. 3.

**Tab. 3:** Grund der Krankenhauseinweisung und Charakteristika der eingeschlossenen Patient:innen unmittelbar vor dem IHCA (*n* = 31)

	Alle Patient:innen (*n* = 31)	QoL Verschlechterung (*n* = 14)	QoL Verbesserung (*n* = 17)	*p*-Wert	Fehlende Daten (*n*/alle)
*Grund der Krankenhauseinweisung*					
Medizinisch	21 (67,7)	13 (92,9)	8 (47,1)	0,009	0/31
Chirurgisch	10 (32,3)	1 (7,1)	9 (52,9)	–	–
*Vigilanz (AVPU)*					–
Wach	29 (93,5)	13 (92,9)	16 (94,1)	1,000	0/31
Reaktion auf Ansprechen	0 (0,0)	0 (0,0)	0 (0,0)		
Reaktion auf Schmerz	0 (0,0)	0 (0,0)	0 (0,0)		
Bewusstlos	2 (6,5)	1 (7,1)	1 (5,9)		
Brustschmerzen	10 (32,3)	6 (42,9)	4 (23,5)	0,441	–
Sauerstoffgabe	4 (12,9)	2 (14,3)	2 (11,8)	1,000	–
*Herzrhythmus*					–
Sinusrhythmus	9 (60,0)	5 (100,0)	4 (40,0)	0,117	16/31
Schrittmacherrhythmus	4 (26,7)	0 (0,0)	4 (40,0)		
Vorhofflimmern	2 (13,3)	0 (0,0)	2 (20,0)		
*Vital- und Laborparameter*					
Körpertemperatur (°C)	36,7 ± 0,5	36,6 ± 1,0	36,7 ± 0,6	0,788	17/31
Herzfrequenz (Schläge/min)	74 ± 20,9	65 ± 14,0	80 ± 22,9	0,097	8/31
Sauerstoffsättigung (%)	95,5 ± 2,4	96,0 ± 2,8	95,3 ± 2,5	0,761	23/31
Blutdruck, systolisch (mm Hg)	129 ± 22,6	131 ± 17,4	128 ± 26,8	0,709	5/31
Blutdruck, diastolisch (mm Hg)	72 ± 13,5	77 ± 12,1	67 ± 13,4	0,058	5/31
Blutdruck, MAD (mm Hg)	91 ± 14,3	97 ± 13,1	85 ± 13,4	0,030	5/31
Letzter Hämoglobinwert (g/l)	124 (69–172)	143 (103–158)	110 (69–172)	0,017	3/31
Erster Hämoglobinwert bei ICU Aufnahme (g/l)	131 (50–165)	144 (93–159)	121 (50–165)	0,045	0/31

*OHCA* „out-of-hospital cardiac arrest“ (präklinischer Herz-Kreislauf-Stillstand), *IHCA* „in-hospital cardiac arrest“ (innerklinischer Herz-Kreislauf-Stillstand), *AVPU* „alert, verbal response, painful stimuli, unresponsive“ (Schema zur Vilgilanzbeurteilung), *MAD* mittlerer arterieller Druck

*Daten angegeben als Mittelwert ± Standardabweichung, Median (Minimum bis Maximum) oder Anzahl (*n*) der Patient:innen (Anteil [%] in Klammern)

Die Daten zu den Vitalparametern werden im Zusatzmaterial online: Tabelle E2 aufgeführt.

Von 31 Patient:innen erlitten 15 (48 %) einen IHCA innerhalb der Kernarbeitszeit (Zusatzmaterial online: Tabelle E3).

Die Ätiologie des IHCA war überwiegend kardial (*n* = 27, 87 %). In einem Fall wurden respiratorische oder medizinische Gründe und in 3 Fällen sonstige Gründe angegeben. Die Zeiten für die kardiopulmonale Reanimation, die Anzahl der Schocks, die verabreichten Medikamente, die Verwendung von mechanischen Reanimationshilfen und das Atemwegsmanagement sind in im Zusatzmaterial online: Tabelle E3 angeführt.

### Lebensqualität nach einem IHCA

Die mittels EQ-5D-5L erhobene gesundheitsbezogene Lebensqualität war bei den überlebenden Patient:innen vor und nach dem IHCA vergleichbar (EQ-5D-5L Utilities 0,79 vs. 0,78, *p* = 0,567) und EQ-5D-5L VAS-Score (Tab. 4). Eine Verbesserung der Lebensqualität nach dem IHCA war mit einer chirurgischen Aufnahmediagnose assoziiert (*p* = 0,009; Tab. 3). Die Daten zur Versorgung nach IHCA sind im Zusatzmaterial online: Tabellen E3 und E4 angegeben.

**Tab. 4:** Lebensqualität vor und nach IHCA (*n* = 31)

EQ-5D-5L Modalitäten	EQ-5D-5L vor IHCA (*n* = 31)	EQ-5D-5L nach IHCA (*n* = 31)	*p*-Wert	Fehlende Daten (*n*/alle)
*EQ-5D-5L, Nutzwert*	0,793 ± 0,312	0,778 ± 0,285	0,567	1/31
*EQ-5D-5L, VAS-Wert*	68,3 ± 26,7	67,5 ± 22,4	0,896	1/31
*EQ-5D-5L VAS-Wert-Kategorie*			–	1/31
Unter 79	15 (50,0)	17 (56,7)		
Über 80	15 (50,0)	13 (43,3)		
*Mobilität*			0,004	1/31
Keine Probleme	14 (46,7)	13 (43,3)		
Leichte Probleme	8 (26,7)	6 (20,0)		
Mäßige Probleme	4 (13,3)	6 (20,0)		
Große Probleme	3 (10,0)	5 (16,7)		
Nicht möglich	1 (3,3)	0 (0,0)		
*Für sich selbst sorgen*			0,248	1/31
Keine Probleme	25 (83,3)	24 (83,3)		
Leichte Probleme	1 (3,3)	0 (0,0)		
Mäßige Probleme	2 (6,7)	4 (13,3)		
Große Probleme	1 (3,3)	1 (3,3)		
Nicht möglich	1 (3,3)	0 (0,0)		
*Schmerzen/körperliche Beschwerden*			0,174	1/31
Keine Probleme	16 (53,3)	11 (36,7)		
Leichte Probleme	7 (23,3)	11 (36,7)		
Mäßige Probleme	4 (13,3)	4 (13,3)		
Große Probleme	1 (3,3)	3 (10,0)		
Extreme Probleme	2 (6,7)	1 (3,3)		
*Alltägliche Tätigkeiten*			0,056	1/31
Keine Probleme	10 (33,3)	11 (36,7)		
Leichte Probleme	7 (23,3)	5 (16,7)		
Mäßige Probleme	9 (30,0)	11 (36,7)		
Große Probleme	2 (6,7)	2 (6,7)		
Nicht möglich	2 (6,7)	1 (3,3)		
*Angst/Depression*			0,039	1/31
Keine Probleme	20 (66,7)	18 (60,0)		
Leichte Probleme	4 (13,3)	4 (13,3)		
Mäßige Probleme	5 (16,7)	6 (20,0)		
Große Probleme	1 (3,3)	2 (6,7)		
Extreme Probleme	0 (0,0)	0 (0,0)		

*IHCA* „in-hospital cardiac arrest“ (innerklinischer Herzstillstand), *VAS* visuelle Analogskala

*Daten angegeben als Mittelwert ± Standardabweichung oder Anzahl(*n*) der Patient/innen (Anteil [%] in Klammern)

Allerdings berichteten die Überlebenden nach dem IHCA vermehrt Probleme hinsichtlich Mobilität und zeigten erhöhte Angst- und Depressionswerte. Der Vergleich aller EQ-5D-5L-Modalitäten ist im Zusatzmaterial online: Abb. E1–E5 dargestellt.

## Diskussion

In dieser prospektiven Erhebung der Lebensqualität zeigte sich bei 31 Überlebenden eines IHCA eine durchwegs gute gesundheitsbezogene Lebensqualität vor und nach dem IHCA. Interessanterweise berichteten die Überlebenden nach dem IHCA über eine eingeschränkte Mobilität sowie über verstärkte Ängste und Depressionen. Dies steht im Gegensatz zu einer rezenten schwedischen Arbeit, die zwar eine eingeschränkte Mobilität bestätigen, jedoch keine verstärkte Angst nach IHCA zeigen konnten [31].

Unsere Überlebensrate von 9 % in 11 Jahren ist im Vergleich zu neueren Metaanalysen mit gepoolten Überlebensraten zwischen 13 % (95 %-KI 6–29 %) und 15 % (95 %-KI 12–18 %) niedriger [11, 29]. Ursächlich dafür könnte der hohe Anteil schwer kranker Patient:innen mit SAPS III Scores > 50 in der vorliegenden Kohorte sein. Dies gilt insbesondere, da Alter und Komorbiditäten wie eine schwer ausgeprägte COPD, eine zirrhotische Lebererkrankung, eine chronische Nierenerkrankung und eine Herzinsuffizienz nachweislich die Überlebensrate verschlechtern [30]. Die oben erwähnten Metaanalysen wiesen jedoch eine recht große statistische Heterogenität auf, wobei eine von ihnen nur über kurzfristige Outcomes wie die Wiedererlangung des Spontankreislaufs (ROSC) und die Entlassung aus dem Krankenhaus berichtete. Darüber hinaus wurde gezeigt, dass Patient:innen mit einem altersangepassten Charlson Comorbidity Index von 5 bis 7 Punkten eine Chance von nur 10 % für das Überleben eines IHCA haben [30]. In unserer Kohorte summierte sich der altersangepasste Charlson Comorbidity Index auf ähnliche Werte von 6 Punkten (nichtgezeigte Daten).

Die 30-Tage-Überlebensrate in der oben genannten Studie (24 %) ist vergleichbar mit der vorhergesagten Überlebensrate auf der Intensivstation bei der Berechnung des SAPS III Scores bei unseren Patient:innen. Unsere Sterblichkeitsrate von 10 % an einem Stichtag entspricht klarerweise nicht der Mortalität auf der Intensivstation oder nach einem Jahr.

Da die Lebensqualität zur Prognoseabschätzung nach einem IHCA immer wichtiger wird, könnte die bloße Betrachtung der Überlebenden die individuellen Auswirkungen eines IHCA unterschätzen. Eine kürzlich durchgeführte schwedische Studie mit 1278 IHCA-Überlebenden bestätigte eine gute Lebensqualität i. Allg. und zeigte eine Verschlechterung der Lebensqualität mit zunehmender Anzahl von Komorbiditäten [31]. In unserer Arbeit berichteten die Überlebenden nach einem IHCA vermehrt über Mobilitätsprobleme und zeigten erhöhte Angst- und Depressionswerte, obwohl die Lebensqualität i. Allg. als gut beschrieben wurde. Eine mögliche Erklärung dafür könnten Vorerkrankungen sein, welche nach dem IHCA zu der funktionellen Verschlechterung beitragen. Dies deckt sich z. T. mit der bereits zitierten Arbeit, in der gezeigt wurde, dass Überlebende mit einer oder mehreren Komorbiditäten mehr Schwierigkeiten in der Bewältigung des täglichen Lebens zeigten. Im Gegensatz zu uns konnten Israelsson et al. jedoch nicht mehr Probleme mit Ängsten/Depressionen feststellen [33]. Diese Divergenz der Studienergebnisse könnte z. T. auf eine limitierte Vergleichbarkeit unterschiedlicher Messinstrumente zurückzuführen sein [32–36]. Zudem wurde der EQ-5D-5L weder für Überlebende eines IHCA entwickelt, geschweige denn dafür validiert.

Patient:innen mit einer Abnahme der Lebensqualität wurden häufiger aufgrund kardiologischer Diagnosen (z. B. Myokardinfarkt, Herzrhythmusstörungen usw.) aufgenommen als Patient:innen mit einer Verbesserung in der Lebensqualität. Dieser Unterschied lässt sich durch die komplexen Vorerkrankungen bei den kardiologischen Patient:innen im Vergleich zu chirurgischen Patient:innen erklären. Darüber hinaus wurden zwei Drittel unserer Patient:innen (22, 71 %) ursprünglich wegen einer herzchirurgischen oder kardialen (i. e. nichtchirurgischen Diagnose) vorstellig und repräsentieren die Hauptkategorie der IHCA-Überlebenden. Dieses deckt sich mit der Literatur; Bergum et al. berichteten über kardiale Ursachen für IHCA in 60 % aller Fälle, wobei nichtschockbare Rhythmen in der Studienpopulation dominierten [37].

Die Literatur hinsichtlich gesundheitsbezogener Lebensqualität nach einem IHCA wächst stetig, wobei der Großteil der Daten nach wie vor von Patient:innen mit einem OHCA stammt. In Übereinstimmung mit dieser Arbeit zeigte eine aktuelle finnische Arbeit die Erhaltung einer guten Lebensqualität nach IHCA. Diese Patient:innen berichteten über eine etwas höhere Lebensqualität nach dem IHCA. Diese könnte darauf zurückzuführen sein, dass die Werte des Charlson Comorbidity Index niedriger waren als in unserer Kohorte. Zudem erlitt in der finnischen Arbeit nur ein Drittel der 55 Patient:innen einen IHCA, der Rest einen OHCA [38]. Frühere Untersuchungen haben gezeigt, dass Patient:innen mit Kammerflimmern als Initialrhythmus im Fall eines OHCA die besten Überlebenschancen und ein gutes Outcome haben [39]. Dies deckt sich mit den jüngsten Arbeiten zum IHCA [39]. Im Gegensatz dazu fanden wir keine Unterschiede in der Lebensqualität in Abhängigkeit vom Initialrhythmus.

Risikofaktoren für ein schlechteres Outcome oder Prädiktoren für einen IHCA konnten in dieser Studie nicht detektiert werden.

### Limitationen

Aufgrund fehlender Follow-up-Daten konnte keine Ein-Jahres-Mortalität berechnet werden. Die nach Stichtag berechnete Mortalität könnte somit Patient:innen verpasst haben, die zwar den IHCA ein Jahr, aber nicht bis zum Stichtag überlebt haben. Weiters könnten Patient:innen übersehen worden sein, die aufgrund einer stark eingeschränkten Lebensqualität nicht in der Lage waren, den Fragebogen zu beantworten. Dies könnte zu einer Überschätzung der Lebensqualität geführt haben. Darüber hinaus ist es schwierig, die IHCA-bedingte Lebensqualität von einer potenziellen Einschränkung der Lebensqualität aufgrund der Grunderkrankung zu unterscheiden. Auch wird das medizinische Notfallteam kaum zu Patient:innen mit HKS alarmiert, die in den Notaufnahmen, Intensivstationen und den OPs auftreten. Dies könnte die tatsächliche Inzidenz der IHCA verringert haben.

## Schlussfolgerungen

Trotz den Errungenschaften der modernen Medizin überlebten in den letzten 11 Jahren am Universitätsklinikum der Medizinischen Universität Innsbruck nur 1 von 10 Patient:innen einen IHCA. Die Überlebenden wiesen eine Einschränkung der Mobilität sowie erhöhte Angst- und Depressionswerte auf. Gleichzeitig wurde die Lebensqualität jedoch insgesamt noch als sehr gut empfunden. Da keine prädiktiven Faktoren für einen IHCA identifiziert werden konnten, gilt aktuell der Fokus auf den veränderbaren Elemente in der Überlebenskette eines IHCA: frühzeitige Erkennung, rasche Aktivierung des Notfallteams und hochqualitative Wiederbelebung. Um die Überlebensrate nach einem IHCA zu steigern, bedarf es einer effektiven Prävention. Zukünftige Studien sollten mit größerem Stichprobenumfang potenziell modifizierbare Faktoren erheben. Darüber hinaus müssen zur Abschätzung der Folgen eines IHCA verfügbare Instrumente zur Bewertung der Lebensqualität angepasst werden.

## Fazit für die Praxis

Trotz der Errungenschaften der modernen Medizin überlebten nur 1 von 10 Patient:innen einen IHCA. Die Überlebenden wiesen eine Einschränkung der Mobilität sowie verstärkte Angstzustände und Depressionen auf. Die Lebensqualität der IHCA-Überlebenden war vor und nach dem Herz-Kreislauf-Stillstand vergleichbar gut.

Künftige Studien mit größeren Patientenstichproben sollten sich auf potenziell modifizierbare Faktoren, die die Folgen eines IHCA verhindern und begrenzen können, konzentrieren. Darüber hinaus sollte die Forschung über die Folgen eines IHCA die verfügbaren Instrumente zur Bewertung der Lebensqualität einbeziehen.

### Supplementary Information

Tabellen  Tabelle E1. STROBE Statement – Checkliste der Punkte, die in Berichten über Beobachtungsstudien enthalten sein sollten; Tabelle E2. Vitalwerte der Patient:innen 1 und 2 Tage vor IHCA (n = 31); Tabelle E3. Reanimationsdaten der Studienpopulation (n=31); Tabelle E4. Post-Reanimationsversorgung der Studienpopulation (n=31); Tabelle E5. Daten der Studienpopulation während der Intensivbehandlung (n=31); Abbildung E1. Lebensqualität vor (rot) and nach IHCA (blau): Mobilität; Abbildung E2. Lebensqualität vor (rot) and nach IHCA (blau): Selbstfürsorge; Abbildung E3. Lebensqualität vor (rot) and nach IHCA (blau): Schmerzen und körperliche Beschwerden; Abbildung E4. Lebensqualität vor (rot) and nach IHCA (blau): Alltägliche Tätigkeiten; Abbildung E5. Lebensqualität vor (rot) and nach IHCA (blau): Angst und Niedergeschlagenheit.

## Data Availability

Die im Rahmen der aktuellen Studie verwendeten und analysierten Datensätze sind auf Anfrage bei den Autor:innen erhältlich.

## Abkürzungen

CPR: „Cardiopulmonary resuscitation“ (Herz-Lungen-Wiederbelebung)
ICU: „Intensive care unit“ (Intensivstation)
IHCA: „In-hospital cardiac arrest“ (innerklinischer Herzstillstand)
ILCOR: International Liaison Committee on Resuscitation
QoL: „Quality of life“ (Lebensqualität)
ROSC: „Return of spontaneous circulation“ (Wiedererlangung eines Spontankreislaufs)
SAPS III: Simplified Acute Physiology Score III
VAS: Visuelle Analogskala („visual analogue scale“)

## References

[1] Mehra R (2007). Global public health problem of sudden cardiac death. J Electrocardiol.

[2] Gräsner J-T, Herlitz J, Tjelmeland IBM, Wnent J, Masterson S, Lilja G (2021). Epidemiologie des Kreislaufstillstands in Europa. Notfall Rettungsmed.

[3] Hodgetts TJ, Kenward G, Vlackonikolis I, Payne S, Castle N, Crouch R (2002). Incidence, location and reasons for avoidable in-hospital cardiac arrest in a district general hospital. Resuscitation.

[4] Skogvoll E, Isern E, Sangolt GK, Gisvold SE (1999). In-hospital cardiopulmonary resuscitation. 5 years’ incidence and survival according to the Utstein template. Acta Anaesthesiol Scand.

[5] Edgren E, Hedstrand U, Kelsey S, Sutton-Tyrrell K, Safar P (1994). Assessment of neurological prognosis in comatose survivors of cardiac arrest. BRCT I Study Group. Lancet.

[6] Haywood K, Whitehead L, Nadkarni VM, Achana F, Beesems S, Böttiger BW (2018). COSCA (core outcome set for cardiac arrest) in adults: an advisory statement from the international liaison committee on resuscitation. Circulation.

[7] van Gijn MS, Frijns D, van de Glind EM, van Munster BC, Hamaker ME (2014). The chance of survival and the functional outcome after in-hospital cardiopulmonary resuscitation in older people: a systematic review. Age Ageing.

[8] van de Glind EM, van Munster BC, van de Wetering FT, van Delden JJ, Scholten RJ, Hooft L (2013). Pre-arrest predictors of survival after resuscitation from out-of-hospital cardiac arrest in the elderly a systematic review. BMC Geriatr.

[9] Andersen LW, Holmberg MJ, Berg KM, Donnino MW, Granfeldt A (2019). In-hospital cardiac arrest: a review. JAMA.

[10] Fuchs A, Käser D, Theiler L, Greif R, Knapp J, Berger-Estilita J (2021). Survival and long-term outcomes following in-hospital cardiac arrest in a Swiss university hospital: a prospective observational study. Scand J Trauma Resusc Emerg Med.

[11] Schluep M, Gravesteijn BY, Stolker RJ, Endeman H, Hoeks SE (2018). One-year survival after in-hospital cardiac arrest: a systematic review and meta-analysis. Resuscitation.

[12] Gottschalk A, Burmeister MA, Freitag M, Cavus E, Standl T (2002). Influence of early defibrillation on the survival rate and quality of life after CPR in prehospital emergency medical service in a German metropolitan area. Resuscitation.

[13] Spearpoint KG, McLean CP, Zideman DA (2000). Early defibrillation and the chain of survival in ‘in-hospital’ adult cardiac arrest; minutes count. Resuscitation.

[14] Soar J, Berg KM (2021). Early epinephrine administration for cardiac arrest. JAMA Netw Open.

[15] Okubo M, Komukai S, Callaway CW, Izawa J (2021). Association of timing of epinephrine administration with outcomes in adults with out-of-hospital cardiac arrest. JAMA Netw Open.

[16] Ran L, Liu J, Tanaka H, Hubble MW, Hiroshi T, Huang W (2020). Early administration of adrenaline for out-of-hospital cardiac arrest: a systematic review and meta-analysis. J Am Heart Assoc.

[17] Debaty G, Shin SD, Metzger A, Kim T, Ryu HH, Rees J (2015). Tilting for perfusion: head-up position during cardiopulmonary resuscitation improves brain flow in a porcine model of cardiac arrest. Resuscitation.

[18] Ryu HH, Moore JC, Yannopoulos D, Lick M, McKnite S, Shin SD (2016). The effect of head up cardiopulmonary resuscitation on cerebral and systemic hemodynamics. Resuscitation.

[19] Tan YK, Han MX, Tan BY, Sia CH, Goh CXY, Leow AS (2022). The role of head-up cardiopulmonary resuscitation in sudden cardiac arrest: a systematic review and meta-analysis. Ann Transl Med.

[20] Amacher SA, Bohren C, Blatter R, Becker C, Beck K, Mueller J (2022). Long-term survival after out-of-hospital cardiac arrest: a systematic review and meta-analysis. JAMA Cardiol.

[21] Buanes EA, Heltne JK (2014). Comparison of in-hospital and out-of-hospital cardiac arrest outcomes in a Scandinavian community. Acta Anaesthesiol Scand.

[22] Høybye M, Stankovic N, Holmberg M, Christensen HC, Granfeldt A, Andersen LW (2021). In-hospital vs. out-of-hospital cardiac arrest: patient characteristics and survival. Resuscitation.

[23] Allencherril J, Lee PYK, Khan K, Loya A, Pally A (2022). Etiologies of in-hospital cardiac arrest: a systematic review and meta-analysis. Resuscitation.

[24] Moskowitz A, Holmberg MJ, Donnino MW, Berg KM (2018). In-hospital cardiac arrest: are we overlooking a key distinction?. Curr Opin Crit Care.

[25] Nolan JP, Berg RA, Andersen LW, Bhanji F, Chan PS, Donnino MW (2019). Cardiac Arrest and Cardiopulmonary Resuscitation Outcome Reports: Update of the Utstein Resuscitation Registry Template for In-Hospital Cardiac Arrest: A Consensus Report From a Task Force of the International Liaison Committee on Resuscitation (American Heart Association, European Resuscitation Council, Australian and New Zealand Council on Resuscitation, Heart and Stroke Foundation of Canada, InterAmerican Heart Foundation, Resuscitation Council of Southern Africa, Resuscitation Council of Asia). Circulation.

[26] Soar J, Böttiger BW, Carli P, Couper K, Deakin CD, Djärv T (2021). European Resuscitation Council Guidelines 2021: Adult advanced life support. Resuscitation.

[27] EuroQol—a new facility for the measurement of health-related quality of life. Health policy (Amsterdam, Netherlands). 1990;16(3):199–208. 10.1016/0168-8510(90)90421-9. 10.1016/0168-8510(90)90421-9 10109801

[28] Ludwig K, von der Schulenburg JM, Greiner W (2018). German Value Set for the EQ-5D-5L. PharmacoEconomics.

[29] Zhu A, Zhang J (2016). Meta-analysis of outcomes of the 2005 and 2010 cardiopulmonary resuscitation guidelines for adults with in-hospital cardiac arrest. Am J Emerg Med.

[30] Piscator E, Hedberg P, Göransson K, Djärv T (2016). Survival after in-hospital cardiac arrest is highly associated with the Age-combined Charlson Co-morbidity Index in a cohort study from a two-site Swedish University hospital. Resuscitation.

[31] Israelsson J, Koistinen L, Årestedt K, Rooth M, Bremer A (2023). Associations between comorbidity and health-related quality of life among in-hospital cardiac arrest survivors—A cross-sectional nationwide registry study. Resuscitation.

[32] Meaney PA, Bobrow BJ, Mancini ME, Christenson J, de Caen AR, Bhanji F (2013). Cardiopulmonary resuscitation quality: [corrected] improving cardiac resuscitation outcomes both inside and outside the hospital: a consensus statement from the American Heart Association. Circulation.

[33] Elliott VJ, Rodgers DL, Brett SJ (2011). Systematic review of quality of life and other patient-centred outcomes after cardiac arrest survival. Resuscitation.

[34] Beesems SG, Wittebrood KM, de Haan RJ, Koster RW (2014). Cognitive function and quality of life after successful resuscitation from cardiac arrest. Resuscitation.

[35] Smith K, Andrew E, Lijovic M, Nehme Z, Bernard S (2015). Quality of life and functional outcomes 12 months after out-of-hospital cardiac arrest. Circulation.

[36] Haydon G, van der Riet P, Maguire J (2017). Survivors’ quality of life after cardiopulmonary resuscitation: an integrative review of the literature. Scand J Caring Sci.

[37] Bergum D, Nordseth T, Mjølstad OC, Skogvoll E, Haugen BO (2015). Causes of in-hospital cardiac arrest—incidences and rate of recognition. Resuscitation.

[38] Hellevuo H, Sainio M, Huhtala H, Olkkola KT, Tenhunen J, Hoppu S (2018). Good quality of life before cardiac arrest predicts good quality of life after resuscitation. Acta Anaesthesiol Scand.

[39] Schluep M, Hoeks SE, Blans M, van den Bogaard B, Koopman-van Gemert A, Kuijs C (2021). Long-term survival and health-related quality of life after in-hospital cardiac arrest. Resuscitation.

