# Supplementary material for: Quality of life after in-hospital cardiac arrest: An 11-year experience from an university center
Source: Anaesthesiologie. 2024 May 31;73(7):454–61. [Article in German] doi: 10.1007/s00101-024-01423-3 (PMC11222208; doi:10.1007/s00101-024-01423-3)

Lebensqualität nach innerklinischem Herzstillstand
11-Jahres-Ergebnisse einer Universitätsklinik

Ergänzende Unterlagen

Inhaltsverzeichnis

- **Tabelle E1.** STROBE Statement – Checkliste der Punkte, die in Berichten über Beobachtungsstudien enthalten sein sollten
- **Tabelle E2.** Vitalwerte der Patient:innen 1 und 2 Tage vor IHCA (n = 31)
- **Tabelle E3.** Reanimationsdaten der Studienpopulation (n=31)
- **Tabelle E4.** Post-Reanimationsversorgung der Studienpopulation (n=31)
- **Tabelle E5.** Daten der Studienpopulation während der Intensivbehandlung (n=31)
- **Abbildung E1.** Lebensqualität vor (rot) and nach IHCA (blau): Mobilität.
- **Abbildung E2.** Lebensqualität vor (rot) and nach IHCA (blau): Selbstfürsorge.
- **Abbildung E3.** Lebensqualität vor (rot) and nach IHCA (blau): Schmerzen und körperliche Beschwerden.
- **Abbildung E4.** Lebensqualität vor (rot) and nach IHCA (blau): Alltägliche Tätigkeiten.
- **Abbildung E5.** Lebensqualität vor (rot) and nach IHCA (blau): Angst und Niedergeschlagenheit.

Tabelle E1. STROBE Statement – Checkliste der Punkte, die in Berichten über Beobachtungsstudien enthalten sein sollten

No.	Merkmal	Empfehlung	Seite
	**Titel and Abstract**		
1		(a) Machen Sie das Studiendesign im Titel oder Abstract kenntlich, indem Sie dafür einen allgemein gebräuchlichen Begriff verwenden	1
		(b) Verfassen Sie für das Abstract eine aussagefähige und ausgewogene Zusammenfassung dessen, was in der Studie gemacht wurde und was herausgefunden wurde	1
	**Einleitung**		
2	Hintergrund/ Rationale	Erläutern Sie den wissenschaftlichen Hintergrund und die Rationale für die vorgestellte Studie	2
3	Zielsetzungen	Geben Sie alle spezifischen Zielsetzungen einschließlich der (vorab festgelegten) Hypothesen an	2
	**Methoden**		
4	Studiendesign	Beschreiben Sie die wichtigsten Elemente des Studiendesigns möglichst früh im Artikel	2-3
5	Rahmen	Beschreiben Sie den Rahmen (Setting) und Ort der Studie und machen Sie relevante zeitliche Angaben, einschließlich der Zeiträume der Rekrutierung, der Exposition, der Nachbeobachtung und der Datensammlung	2-3
6	Studien-teilnehmer	(a) Kohortenstudie – Geben Sie die Einschlusskriterien, die Herkunft der Teilnehmer sowie die Methoden ihrer Auswahl an; beschreiben Sie die Methoden der Nachbeobachtung Fallkontrollstudie – Geben Sie die Einschlusskriterien und die Herkunft der Fälle und Kontrollen an sowie die Methoden, mit denen die Fälle erhoben und die Kontrollen ausgewählt wurden. Geben Sie eine Begründung (Rationale) für die Auswahl der Fälle und Kontrollen<br>Querschnittsstudie – Geben Sie die Einschlusskriterien, die Herkunft der Teilnehmer sowie die Methoden ihrer Auswahl an	2-3
7	Variablen	Definieren Sie eindeutig alle Zielgrößen, Expositionen, Prädiktoren, möglichen Confounder und Effektmodifikatoren; geben Sie gegebenenfalls Diagnosekriterien an	2-3
8*	Datenquellen / Messmethoden	Geben Sie für jede in der Studie wichtige Variable die Datenquellen an und erläutern Sie die verwendeten Bewertungs- bzw. Messmethoden. Beschreiben Sie die Vergleichbarkeit der Messmethoden, wenn es mehr als eine Gruppe gibt	2-3
9	Bias	Beschreiben Sie, was unternommen wurde, um möglichen Ursachen von Bias zu begegnen	2-3
10	Studiengröße	Erklären Sie, wie die Studiengröße ermittelt wurde	NA
11	Quantitative Variablen	Erklären Sie, wie in den Auswertungen mit quantitativen Variablen umgegangen wurde Wenn nötig, beschreiben Sie, wie Kategorien (Gruppierungen) gebildet wurden und warum	2-3
12	Statistische Methoden	(a) Beschreiben Sie alle statistischen Methoden, einschließlich der Methoden, die für die Kontrolle von Confounding verwendet wurden	3
		(b) Beschreiben Sie Verfahren, mit denen Subgruppen und Interaktionen untersucht wurden	3
		(c) Erklären Sie, wie mit fehlenden Daten umgegangen wurde	3
		(d) Kohortenstudie – Erklären Sie gegebenenfalls wie mit dem Problem des vorzeitigen Ausscheidens aus der Studie („loss to follow-up“) umgegangen wurde<br>Fallkontrollstudie – Beschreiben Sie gegebenenfalls wie das Matching (Paarbildung) von Fällen und Kontrollen bei der Auswertung berücksichtigt wurde<br>Querschnittsstudie – Beschreiben Sie gegebenenfalls die Auswertungsmethoden, die die gewählte Strategie zur Stichprobenauswahl (Sampling strategy) berücksichtigen	NA
		(e) Beschreiben Sie vorgenommene Sensitivitätsanalysen	NA
	**Ergebnisse**		
13*	Teilnehmer	(a) Geben Sie die Anzahl der Teilnehmer während jeder Studienphase an, z. B. die Anzahl der Teilnehmer, die potenziell geeignet waren, die auf Eignung untersucht wurden, die als geeignet bestätigt wurden, die tatsächlich an der Studie teilgenommen haben, deren Nachbeobachtung abgeschlossen wurde und deren Daten ausgewertet wurden	3-4
		(b) Geben Sie die Gründe für die Nicht-Teilnahme in jeder Studienphase an	Fig. 1
		(c) Erwägen Sie die Darstellung in einem Flussdiagramm	Fig. 1

14*	Deskriptive Daten	(a) Beschreiben Sie Charakteristika der Studienteilnehmer (z. B. demographische, klinische und soziale Merkmale) sowie Expositionen und mögliche Confounder	Tab.
		(b) Geben Sie für jede Variable die Anzahl der Teilnehmer mit fehlenden Daten an	Tab.
		(c) Kohortenstudie – Fassen Sie die Nachbeobachtungszeit zusammen (z. B. Mittelwert und Gesamtzeitraum)	Tab.
15*	Ergebnisdaten	Kohortenstudie – Berichten Sie über die Anzahl der Zielereignisse oder statistische Maßzahlen (z. B. Mittelwert und Standardabweichung) im zeitlichen Verlauf	Tab.
16	Hauptergebnisse	(a) Geben Sie die unadjustierten Schätzwerte an und gegebenenfalls auch die Schätzwerte, in denen Adjustierungen für die Confounder vorgenommen wurden sowie deren Präzision (z. B. 95%-Konfidenzintervall); machen Sie deutlich, für welche Confounder adjustiert wurde und warum diese berücksichtigt wurden	Tab.
		(b) Wenn stetige Variablen kategorisiert wurden, geben Sie die oberen und unteren Grenzwerte der einzelnen Kategorien an	Tab.
		(c) Wenn relevant, erwägen Sie, für aussagekräftige Zeiträume Schätzwerte relativer Risiken auch als absolute Risiken auszudrücken	NA
17	Weitere Auswertungen	Berichten Sie über weitere vorgenommene Auswertungen, z. B. die Analyse von Subgruppen und Wechselwirkungen (Interaktionen) sowie Sensitivitätsanalyse	4-5
	**Diskussion**		
18	Hauptergebnisse	Fassen Sie die wichtigsten Ergebnisse in Hinsicht auf die Studienziele zusammen	4-5
19	Einschränkungen	Diskutieren Sie die Einschränkungen der Studie und berücksichtigen Sie dabei die Gründe für möglichen Bias oder Impräzision<br>Diskutieren Sie die Richtung sowie das Ausmaß jedes möglichen Bias	5-6
20	Interpretation	Nehmen Sie eine vorsichtige übergreifende Interpretation der Resultate vor und berücksichtigen Sie dabei die Ziele und Einschränkungen der Studie, die Multiplizität der Analysen, die Ergebnisse anderer Studien und andere relevante Evidenz	4-6
21	Übertragbarkeit	Besprechen Sie die Übertragbarkeit (externe Validität) der Studienergebnisse	4-6
	**Zusätzliche Information**		
22	Finanzierung	Geben Sie an, wie die vorliegende Studie finanziert wurde, und erläutern Sie die Rolle der Geldgeber. Machen Sie diese Angaben gegebenenfalls auch für die Originalstudie, auf welcher der vorliegende Artikel basiert	Titelse ite

** Geben Sie diese Informationen jeweils gesondert für Fälle und Kontrollen in Fallkontrollstudien an, und gegebenenfalls für exponierte und nicht-exponierte Gruppen in Kohorten- und Querschnittsstudien

Tabelle E2. Vitalwerte der Patient:innen ein bzw. zwei Tage vor IHCA (n = 31)

	**Alle Patient:innen (n = 31)**	**QoL Verschlechterung (n = 14)**	**QoL Verbesserung (n = 17)**	**p-Wert**	**Fehlende Daten (n/alle)**
**Ein Tag vor IHCA**					
Körperkerntemperatur (°C)	36.7 ±0.5	36.6 ±0.6	36.7 ±0.5	0.788	17/31
Herzfrequenz (Schläge/min)	74 ±20.9	65 ±14.4	80 ±22.9	0.097	8/31
Sauerstoffsättigung (%)	96 ±2.4	96 ±2.8	95 ±2.4	0.761	23/31
Systolischer Blutdruck (mmHg)	103 (103-189)	106 (106-168)	126 (103-189)	0.752	8/31
Diastolischer Blutdruck (mmHg)	70 (49-100)	70 (51-88)	70 (49-100)	0.752	8/31
Mittlerer arterieller Blutdruck (mmHg)	90 (68-118)	89 (69-115)	91 (60-118)	0.411	8/31
**Zwei Tage vor IHCA**					
Körperkerntemperatur (°C)	36.7 ±0.7	37.1±0.6	36.5 ±0.8	0.598	22/31
Herzfrequenz (Schläge/min)	69 ±13.8	64 ±15.1	74 ±11.8	0.145	14/31
Sauerstoffsättigung (%)	95 ±1.9	94 ±0.0	95 ±2.0	0.740	25/31
Systolischer Blutdruck (mmHg)	117 (93-153)	123 (93-153)	114 (100-130)	0.142	13/31
Diastolischer Blutdruck (mmHg)	73 (45-94)	73 (60-94)	69 (45-85)	0.397	13/31
Mittlerer arterieller Blutdruck (mmHg)	85 (71-111)	87 (74-111)	81 (71-107)	0.246	13/31

Daten dargestellt als Mittelwert ± Standardabweichung oder Median (Minimum – Maximum). Abkürzungen: QoL, Lebensqualität/quality of life; IHCA, In-hospital cardiac arrest.

Tabelle E3. Reanimationsdaten der Studienpopulation (n=31)

Klinische Merkmale	Alle Patient:innen (n = 31)	QoL Verschlechterung (n = 14)	QoL Verbesserung (n = 17)	p-Wert	Fehlende Daten (n/alle)
**Uhrzeit des Notfalls**					0/31
Kernarbeitszeit (7:30-16:00)	15 (48.4)	9 (64.3)	6 (35.3)		
Außerhalb der Kernarbeitszeit (16:00-07:30)	16 (51.6)	5 (35.7)	11 (64.7)	0.156	
**Klinische Merkmale bei Kreislaufstillstand**					
Pulsloser Patient	29 (93.5)	13 (92.9)	16 (94.1)	1.000	0/31
Beobachtetes Ereignis (Person / Monitor)	28 (90.3)	14 (100.0)	14 (82.4)	0.232	0/31
Montierter Kreislaufstillstand	16 (51.6)	8 (57.1)	8 (47.1)	0.722	0/31
Herzdruckmassage	29 (93.5)	13 (92.9)	16 (94.1)	1.000	0/31
AED-Anwendung	18 (58.1)	7 (50.0)	11 (64.7)	0.690	0/31
Vorbestehende Sepsis, Hypotension, metastatiertes oder hämatologisches Malignom, Leber- oder Nierenversagen	2 (6.5)	1 (7.1)	1 (5.9)	1.000	0/31
Interner oder externer Cardioverter-Defibrillator	2 (6.5)	0 (0.0)	2 (11.8)	0.488	0/31
**Initialer Rhythmus**					2/31
Kammerflimmern oder -tachykardie	20 (69.0)	9 (69.2)	11 (68.8)		
Pulslose elektrische Aktivität	2 (6.9)	0 (0.0)	2 (12.5)	0.429	
Asystolie	6 (20.7)	4 (30.8)	2 (12.5)		
Unbekannt	1 (3.4)	0 (0.0)	1 (6.3)		
**Aktuelle Diagnose bei Kreislaufstillstand**					0/31
Koronare Herzkrankheit	10 (32.3)	7 (50.0)	3 (17.6)		
Kardiale Erkrankung	10 (32.3)	4 (28.6)	6 (35.3)	0.159	
Andere	11 (35.5)	3 (21.4)	8 (47.1)		
**Reanimationsmaßnahmen (Zeiten)**					0/31
Kollaps bis Start CPR (Minuten)	0 (0-2)	0 (0-2)	0 (0-2)	0.298	
Kollaps bis Start ALS (Minuten)	3 (0-7)	3 (0-5)	4 (0-7)	0.246	
Kollaps bis erste Defibrillation (Minuten)	3 (0-17)	2 (0-17)	3 (1-6)	0.926	
CPR-Dauer (Minuten)	6 (1-61)	7 (1-61)	5 (1-20)	0.273	
**Defibrillation**					0/31
Schockanzahl	22 (71.0)	10 (71.4)	12 (70.6)	1.000	
Durchschnittliche Schockanzahl	1 (0-9)	2 (0-9)	1 (0-3)	0.240	
**Medikation**					0/31
Adrenalin-Gaben	0 (0-8)	0 (0-8)	0 (0-4)	0.652	
Amiodaron-Gaben					
150 mg	3 (9.7)	2 (14.3)	1 (5.9)		
300 mg	4 (12.9)	3 (21.4)	1 (5.9)	0.275	
600 mg	1 (3.2)	1 (7.1)	0 (0.0)		
**Mechanische CPR**					0/31
Nur Thoraxkompressionen	25 (80.6)	9 (64.3)	16 (94.1)	0.067	
Thoraxkompressionen und LUCAS	5 (16.1)	5 (35.7)	0 (0.0)	0.012	
Nur LUCAS	0 (0.0)	0 (0.0)	0 (0.0)		
ECMO	0 (0.0)	0 (0.0)	0 (0.0)		

**Atemwegssicherung**					0/31
Oropharyngeale Atemwegshilfen	2 (6.5)	1 (7.1)	1 (5.9)	1.000	
Beutel-Masken-Beatmung	6 (19.4)	2 (14.3)	4 (23.5)	0.664	
Supraglottische Atemwegshilfen	0 (0.0)	0 (0.0)	0 (0.0)		
Endotrachealtubus	12 (38.7)	7 (50.0)	5 (29.4)	0.288	
Kapnometrie/Kapnografie	12 (38.7)	7 (50.0)	5 (29.4)	0.288	
Farbcodiertes exspiratorisches CO_2 Monitoring	0 (0.0)	0 (0.0)	0 (0.0)		

Daten dargestellt als Median (Minimum – Maximum) oder Anzahl der Patient:innen (%). Bei gleichen Medianwerte werden zur Verdeutlichung Mittelwerte dargestellt.

Abkürzungen: QoL, Lebensqualität/quality of life; CPR, kardiopulmonale Reanimation; ALS, erweiterte lebensrettende Maßnahmen; AED, automatischer externer Defibrillator; ECMO, extrakorporale Membranoxygenierung; LUCAS, Lund University Cardiac Assist System; VAD, Ventricular Assist Device, IHCA, In-hospital cardiac arrest.

Tabelle E4. Post-Reanimationsversorgung der Studienpopulation (n=31)

	Alle Patient:innen (n = 31)	QoL Verschlechterung (n = 14)	QoL Verbesserung (n = 17)	p-Wert	Fehlende Daten (n/alle)
Aktive Temperaturkontrolle nach ROSC	3 (9.7)	2 (14.3)	1 (5.9)	0.576	0/31
Antipyretika nach ROSC	2 (6.5)	1 (7.1)	1 (5.9)	1.000	0/31
**Koronarangiographie**					0/31
Dringlich (innerhalb von 2h nach IHCA)	6 (19.4)	5 (35.7)	1 (5.9)		
Verzögert (während desselben Krankenhausaufenthalts)	7 (22.6)	2 (14.3)	5 (29.4)	0.133	
Keine Koronarangiographie	18 (58.1)	7 (50.0)	11 (64.7)		
**Koronare Reperfusion**					0/31
Perkutane koronare Intervention	7 (22.6)	5 (35.7)	2 (11.8)		
Thrombolyse	2 (6.5)	1 (7.1)	1 (5.9)	0.240	
Keine Intervention	22 (71.0)	8 (57.1)	14 (82.4)		
**Zeitpunkt der koronaren Intervention**					16/31
Peri-arrest	4 (26.7)	4 (50.0)	0 (0.0)		
Innerhalb von 24h nach ROSC	6 (40.0)	2 (25.0)	4 (57.1)	0.119	
Unbekannt	5 (33.3)	2 (25.0)	3 (42.9)		
**Erstes 12-Kanal EKG nach ROSC**					0/31
STEMI	6 (19.4)	5 (35.7)	1 (5.9)		
Ischämische Veränderugnen (kein STEMI)	1 (3.2)	0 (0.0)	1 (5.9)	0.141	
Neuer Linksschenkelblock	1 (3.2)	0 (0.0)	1 (5.9)		
Andere	23 (74.2)	9 (64.3)	14 (82.4)		

Daten dargestellt als Median (Minimum – Maximumbereich) oder Anzahl der Patient:innen (%). Bei gleichen Medianwerte werden zur Verdeutlichung Mittelwerte dargestellt.
Abkürzungen: ROSC, return of spontaneous circulation; IHCA, In-hospital cardiac arrest; Lebensqualität/quality of life; STEMI, ST-Hebungsinfarkt.

Tabelle E5. Daten der Studienpopulation während der Intensivbehandlung (n=31)

	**Alle Patient:innen (n = 31)**	**QoL Verschlechterung (n = 14)**	**QoL Verbesserung (n = 17)**	**p-Wert**	**Fehlende Daten (n/alle)**
Wiederaufnahme auf die Intensivstation	5 (16.1)	1 (7.1)	4 (23.5)	0.344	0/31
Wiederholte Interventionen des innerklinischen Notfallteams	1 (3.2)	0 (0.0)	1 (5.9)	1.000	0/31
Dauer der mechanischen Beatmung (Tage)	0 (0-11), Mean 2	0 (0-11), Mean 2	0 (0-9), Mean 2	0.138	0/31
**Erste arterielle Blutgasanalyse**					
pH	7.32 ±0.17	7.27 ±0.17	7.36 ±0.17	0.034	1/31
Laktat (mg/dl)	22.0 (8.0-126.0)	17.0 (8.0-126.0)	24.5 (10.0-118.0)	0.417	1/31
pCO_2 (mmHg)	41.5 (22.7-97.2)	44.9 (32.8-97.2)	36.5 (22.7-77.4)	0.044	1/31
pO_2 (mmHg)	72.3 (20-341)	80.3 (20-341)	70.9 (27-316)	0.835	1/31
**Erstes Labor nach ROSC**					
Erster pH	7.33 ±0.17	7.27 ±0.17	7.37 ±0.16	0.089	0/31
Zweiter pH*	7.37 ±0.12	7.33 ±0.13	7.39 ±0.10	0.178	4/31
Dritter pH*	7.40 ±0.07	7.39 ±0.08	7.41 ±0.07	0.398	6/31
Vierter pH*	7.39 ±0.07	7.38 ±0.08	7.40 ±0.07	0.336	9/31
Fünfter pH*	7.40 ±0.06	7.39 ±0.05	7.41 ±0.06	0.350	11/31
Initiales Laktat (mg/dl)	22.0 (8.0-126.0)	17.0 (8.0-126.0)	26.0 (10.0-118.0)	0.404	0/31
Zweites Laktat (mg/dl)*	13.4 (6.0-98.0)	14.5 (6.0-89.0)	13.0 (6.0-98.0)	0.406	4/31
Drittes Laktat (mg/dl)*	12.0 (5.0-92.0)	12.2 (6.0-83.0)	12.0 (5.0-92.0)	0.827	6/31
Viertes Laktat (mg/dl)*	16.5 (6.0-84.0)	24.0 (6.0-63.0)	12.0 (6.0-84.0)	0.316	9/31
Fünftes Laktat (mg/dl)*	12.5 (5.0-83.0)	24.0 (6.0-83.0)	11.5 (5.0-74.0)	0.279	11/31
pCO_2 (mmHg)	43.4 ±15.2	49.6 ±16.3	38.2 ±12.6	0.037	0/31
pO_2 (mmHg)	71.7 (20-341)	80.3 (20-341)	70.0 (27-316)	0.843	0/31
Hämoglobin (g/l)	13.1 (5.0-16.5)	14.4 (9.3-15.9)	12.1 (5.0-16.5)	0.045	0/31
Thrombozytenzahl (×109/L)	180 (31-448)	178.5 (101-448)	187 (31-384)	0.708	1/31
Blutzucker (mg/dl)	160 (101-388)	161 (101-388)	150 (107-305)	0.487	0/31

Daten dargestellt als Mittelwert ± Standardabweichung, Median (Minimum – Maximumbereich) oder Anzahl der Patient:innen (%). Bei gleichen Medianwerte werden zur Verdeutlichung Mittelwerte dargestellt. * Werte mit mindestens 1h Abstand zur vorhergehenden Messung. Abkürzungen: QoL, Lebensqualität/quality of life; ROSC, return of spontaneous circulation.

Abbildung E1. Lebensqualität vor (rot) and nach IHCA (blau): Mobilität.

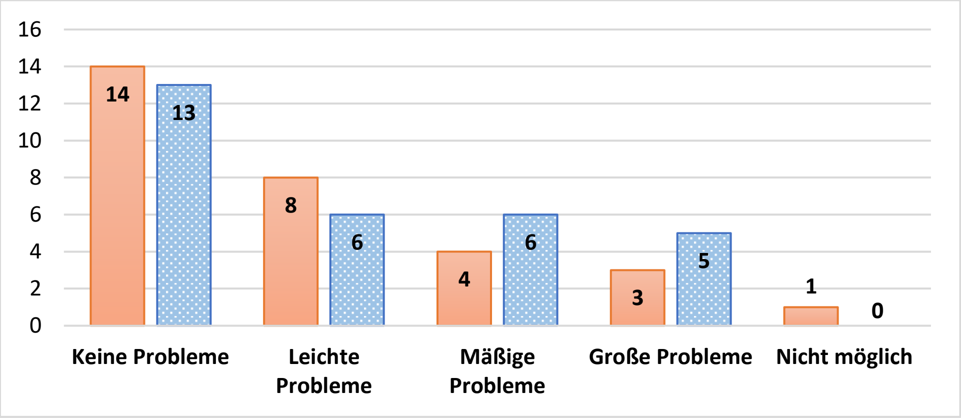

Abbildung E2. Lebensqualität vor (rot) and nach IHCA (blau): Selbstfürsorge.

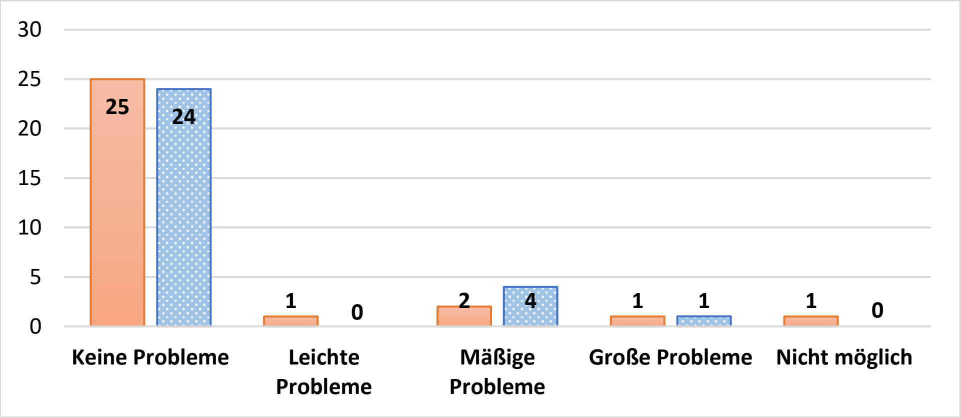

Abbildung E3. Lebensqualität vor (rot) and nach IHCA (blau): Schmerzen und körperliche Beschwerden.

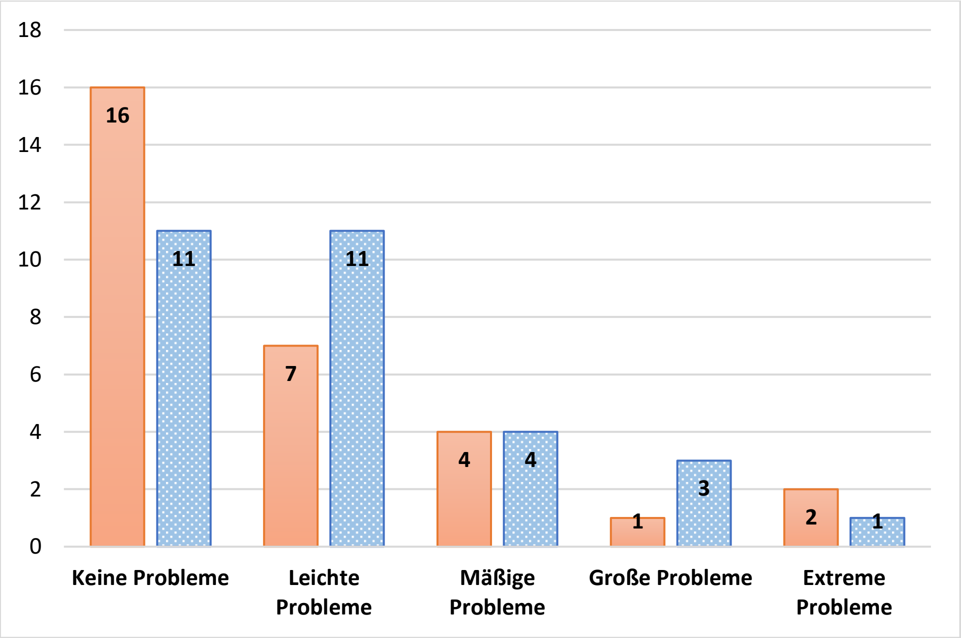

Abbildung E4. Lebensqualität vor (rot) and nach IHCA (blau): Alltägliche Tätigkeiten.

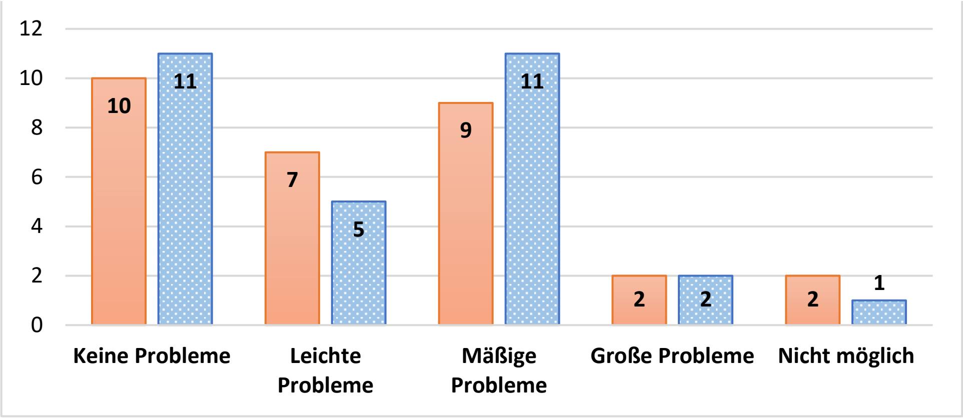

Abbildung E5. Lebensqualität vor (rot) and nach IHCA (blau): Angst / Depression.

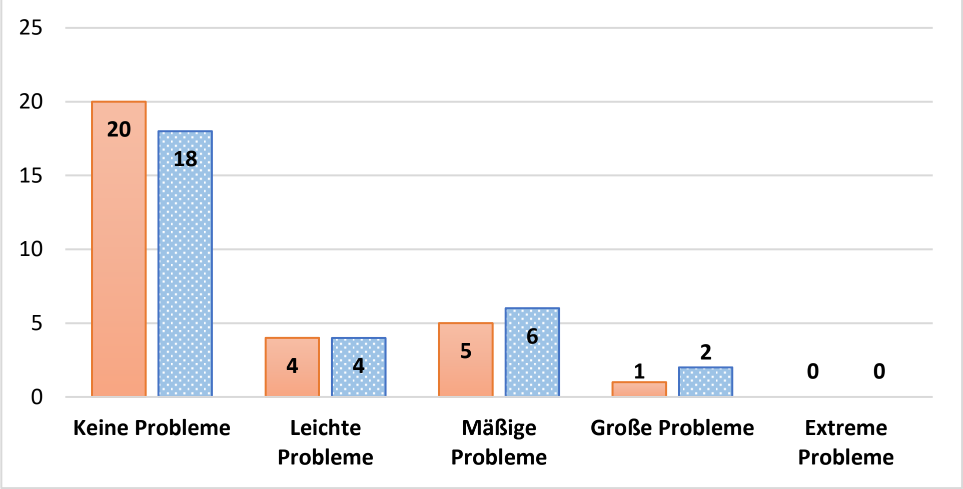

Supplement: Supplementary file 1 — Tabellen Tabelle E1. STROBE Statement – Checkliste der Punkte, die in Berichten über Beobachtungsstudien enthalten sein sollten; Tabelle E2. Vitalwerte der Patient:innen 1 und 2 Tage vor IHCA (n = 31); Tabelle E3. Reanimationsdaten der Studienpopulation (n=31); Tabelle E4. Post-Reanimationsversorgung der Studienpopulation (n=31); Tabelle E5. Daten der Studienpopulation während der Intensivbehandlung (n=31); Abbildung E1. Lebensqualität vor (rot) and nach IHCA (blau): Mobilität; Abbildung E2. Lebensqualität vor (rot) and nach IHCA (blau): Selbstfürsorge; Abbildung E3. Lebensqualität vor (rot) and nach IHCA (blau): Schmerzen und körperliche Beschwerden; Abbildung E4. Lebensqualität vor (rot) and nach IHCA (blau): Alltägliche Tätigkeiten; Abbildung E5. Lebensqualität vor (rot) and nach IHCA (blau): Angst und Niedergeschlagenheit. [file 101_2024_1423_MOESM1_ESM.pdf]
